# Rare *Helicobacter pylori* Virulence Genotypes in Bhutan

**DOI:** 10.1038/srep22584

**Published:** 2016-03-02

**Authors:** Osamu Matsunari, Muhammad Miftahussurur, Seiji Shiota, Rumiko Suzuki, Ratha-korn Vilaichone, Tomohisa Uchida, Thawee Ratanachu-ek, Lotay Tshering, Varocha Mahachai, Yoshio Yamaoka

**Affiliations:** 1 Department of Environmental and Preventive Medicine, Oita University Faculty of Medicine, Yufu, Japan; 2 Gastroentero-Hepatology Division, Department of Internal Medicine, Faculty of Medicine-Institute of Tropical Disease, Airlangga University, Surabaya, Indonesia; 3 Gastroenterology Unit, Department of Medicine, Thammasat University Hospital, Pathumthani, Thailand; 4 Department of Molecular Pathology, Oita University Faculty of Medicine, Yufu, Japan; 5 Department of Surgery, Rajavithi Hospital, Bangkok, Thailand; 6 Department of Surgery, Jigme Dorji Wangchuck National Referral Hospital, Thimphu, Bhutan; 7 GI and Liver Center, Bangkok Medical Center, Bangkok, Thailand; 8 Department of Medicine, Gastroenterology and Hepatology Section, Baylor College of Medicine, Houston, Texas, USA

## Abstract

Both the prevalence of *Helicobacter pylori* infection and the incidence of gastric cancer are high in Bhutan. The high incidence of atrophic gastritis and gastric cancer suggest the phylogeographic origin of an infection with a more virulent strain of *H. pylori*. More than 90% of Bhutanese strains possessed the highly virulent East Asian-type CagA and all strains had the most virulent type of *vacA* (s1 type). More than half also had multiple repeats in East Asian-type CagA, which are rare in other countries and are reported characteristictly found in assciation with atrophic gastritis and gastric cancer consistent with Bhutanese strains having multiple *H. pylori* virulence factors associated with an increase in gastric cancer risk. Phylogeographic analyses showed that most Bhutanese strains belonged to the East Asian population type with some strains (17.5%) sharing East Asian and Amerindian components. Only 9.5% belonged to the European type consistant with *H. pylori* in Bhutan representing an intermediate evolutionary stage between *H. pylori* from European and East Asian countries.

*Helicobacter pylori* is a spiral-shaped, gram-negative bacterial pathogen infecting more than half of the world’s population. *H. pylori* plays a causative role in the pathogenesis of gastritis, peptic ulcer diseases, gastric cancer, and mucosa-associated lymphoid tissue lymphoma^1^,^2^. Although infection with *H. pylori* always results in histologic gastritis, the clinical outcome in different populations varies in relation *H. pylori* virulence, host genetic factors and environmental factors (especially diet)^3^,^4^. The cytotoxin-associated gene A (CagA) and vacuolating cytotoxin (VacA) are the most extensively studied *H. pylori* virulence factors^5^,^6^,^7^,^8^. *cagA*-positive strains are subdivided into an East Asian type and Western type depending on sequences in the 3′ region of the gene^9^,^10^,^11^ which contain a tyrosine phosphorylation site motif Glu-Pro-Ile-Tyr-Ala (EPIYA). The EPIYA motifs can be further subdivided into four distinct peptide segments, EPIYA-A, EPIYA-B, EPIYA-C, and EPIYA-D based on amino acid sequences flanking the EPIYA motif^12^. Most CagA-positive isolates possess EPIYA-A or EPIYA-B segments. The EPIYA-C genotype is characteristic of CagA from Western countries (Western-type CagA), and the EPIYA-D segment is characteristic of East Asian-type CagA^9^,^10^,^11^,^13^. CagA sequence type is based on all the EPIYA segments in the sequence (i.e., ABC, ABCC, ABCCC, etc. for Western-type CagA and ABD, etc. for East Asian-type CagA). The East Asian-type CagA is associated with a more robust mucosal inflammatory reaction than Western-type CagA and epidemiological studies in Thailand, South Korea, and Okinawa, Japan have shown East Asian-type CagA strains correlate with an increased risk of peptic ulcer or gastric cancer compared with those infected with Western-type CagA strains^14^,^15^,^16^.

Different genotypes of *vacA* have been described based on differences in the signal (s) region (s1 and s2) and the middle (m) region (m1 and m2)^6^. In epidemiological studies *vacA* has been classified into subtypes based on the combination of variants in the s and m regions. *In vitro* experiments have shown that s1m1 strains are the most cytotoxic, followed by s1m2 strains; s2m2 strains have no cytotoxic activity and do not produce VacA protein^6^; the s2m1 genotype is rare. Epidemiological studies have shown that infection with the *vacA* s1 or m1 strains correlates with an increased risk for peptic ulcers or gastric cancer compared with those with s2 or m2 strains, especially in Western countries^6^,^17^,^18^,^19^. Importantly, the presence of CagA and VacA are typically linked such that *H. pylori* either produce both or neither protein^7^.

*H. pylori* infection is one of those infectious diseasese that accompanied humans on their journey out of Africa. The diversity within *H. pylori* lineages examined by multi-locus sequence typing (MLST) using seven housekeeping genes^20^,^21^,^22^ identified seven population types based on geographical associations: hpEurope, hpEastAsia, hpAfrica1, hpAfrica2, hpAsia2, hpNEAfrica, and hpSahul^20^,^21^,^22^. Besides possessing East Asian-type cagA^20^,^21^,^22^, hpEastAsia type has been further divided into hspEAsia, hspAmerind, and hspMaori. Studies in Colombia suggested that European strain origin (hpEurope) was associated with premalignant histological lesions compared to infection with African strains (hpAfrica1)^23^,^24^. However no previous studies have focused on the relationship between the phylogeographic origin of *H. pylori* and gastric cancer risk in Asia despite the data regarding the more virulent type of CagA of hpEastAsia origin compared to strains of hpAsia2 and/or hpEurope origins.

Bhutan is a small, landlocked country in South Asia, located at the eastern end of the Himalayas, that shares borders in the south, east, and west with the Republic of India and to the north with the People’s Republic of China. The incidence of gastric cancer is reported to be greater than that of neighboring areas (17.2 cases per 100,000 individuals per year compared with 6.1 cases/100,000 individuals per year in India) (International Agency for Research on Cancer; GLOBOCAN2012, http://globocan.iarc.fr/). In India, Western-type CagA and the hpAsia2, and/or hpEurope types are predominant possibly reflecting the gene flow through Indo-Aryans that replaced endogenous strains in India^20^,^21^,^22^,^25^. We hypothesize that the difference in the incidence of gastric cancer between India and Bhutan could be in part related to differences in phylogeographic origin and virulence of *H. pylori*. In this study, we also examined the *H. pylori* virulence factors in Bhutan and their relationship with histological scores and clinical presentations.

## Results

A total of 209 strains were isolated from *H. pylori*-positive Bhutanese volunteers (98 males, 16–92 years old, mean 35.6 years; 111 females, 16–75 years old, mean 37 years). Of these, 165 strains were isolated from subjects with histological gastritis without peptic ulcers or gastric cancer, 21 from gastric ulcer (GU) patients, 19 from duodenal ulcer (DU) subjects, 1 from a gastric cancer subject, and 3 from subjects with an unclear diagnosis (Table 1). The average age was significantly lower in DU subjects than in subjects with simple *H. pylori* gastritis (mean 29.4 years vs. mean 36.7 years; P = 0.03). The male/female ratio was significantly higher for the GU and DU subjects than for the gastritis subjects (male:female = 16:5 for GU, 13:6 for DU and 67:98 for gastritis; P < 0.002 and P = 0.02, respectively).

### *cagA* genotypes

*cagA* was present in 98.6% (206/209) of *H. pylori* strains cultured (Table 1). The 3 strains negative for *cagA* by PCR showed negative band using the *cag* pathogenicity island (PAI) empty site PCR indicating the presence of at least a partial *cag* PAI; therefore, we considered them *cagA*-undetermined. Two strains were *cagA–*positive based on PCR; however, the *cagA* genotypes were undetermined by sequencing because the primer pair did not amplify the gene. Sequencing showed that among *cagA*-positive strains, the East Asian-type CagA was predominant (189/206, 91.7%); Western-type CagA was found in 7.3% of strains (15/206) Sequencing analysis revealed that only 82 (43.4%) of the 189 East Asian-type CagA strains possessed the typical East Asian genotype (ABD) (Table 2). For Western-type CagA, 13 of 15 had the ABC genotype (86.7%). The two remaining strains were AB- and AC-type.

In addition to the four major segments originally designated as EPIYA-A, -B, -C, and -D (i.e., A, B, C, and D), we previously designated several minor segments, including EPIYA-B’ and –B”^26^. EPIYA segments were classified according to these groups. The AB’BD type was most prominent among the East Asian strains (97/189; 51.3%). This type represents only 0.7% [2/280] of East Asian-type CagA strains deposited in GenBank^26^.

**Figure 1:**
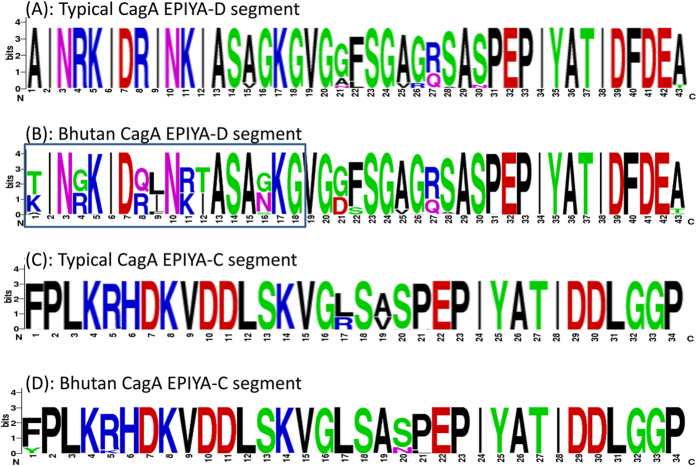
Variation in the CagA amino acid sequence of East Asian-type and Western-type CagA. The target α-EAS sequences “AINRKIDRINKIASAGKG” in EPIYA segment D, based on the sequences of Japanese strains, are shown in frame. A sequence analysis revealed that the typical target sequences in Bhutanese strains was “(T/K)IN(G/R)KID(Q/R)(L/I)N(R/K)(T/I)ASA(G/N)KG,” where (X/Y) means X and Y as the two major amino acids. The sequences of the EPIYA-D segments in Bhutanese strains were highly variable compared to strains deposited in GenBank. In contrast, the East Asian-type and Western-type sequences of EPIYA-C segments in Bhutanese strains were largely identical, similar to the typical sequences of EPIYA-C segments deposited in GenBank. Reference strains used in Fig. 1A (strain name [accession number]) were 103a (AB110966.1), 105a (AB110967.1), 106a (AB110968.1), 108a (AB110969.1), 113b (AB110970.1), 120a (AB110971.1), 122b (AB110972.1), 125b (AB110973.1), 128a (AB110974.1), FJT77 (KF028580.1), 04-518 (AB267252.1), 03-166 (AB267253.1), 04-264 (AB267254.1), THP1477 (AB116744.1), 04-334 (AB267249.1), 03-292 (AB267250.1), 04-366 (AB267251.1), THP1260 (AB116742.1), M3 (AB116740.1), THP463 (AB116735.1), Korea23 (AB057044.1), Korea 12 (AB057043.1), K69 (FJ458129.1), Korea2-3 (AB057040.1), k266 (FJ458163.1), K265 (FJ458162.1), K264 (FJ458161.1), K261 (FJ458158.1), K260 (FJ458157.1), K259 (FJ458156.1). Reference strains used in Fig. 1C (strain name [accession number]) were India41 (AF222807.1), India99 (AF222809.1), OSC40A (EU089774.1), OSC42B (EU089775.1), PCR-156i (EU368669.1), PCR218vi (EU089766.1). RIGLD-OC149 (JX428784.1), SAN53 (EU089771.1), PD682 (EF450167.1), PD636 (EF450165.1), PD308 (EF450162.1), PD488 (EF450161.1), PD537 (EF450160.1), PD501 (EF450159.1), PD351 (EF450158.1), PD348 (EF450157.1), PD6481K (EF450153.1), 216G (GQ899171.1), 1407 (GU143415.1), HPI-14 (FJ849792.1), HPI-13 (FJ849791.1), HPI-11 (FJ849789.1), USA2791 (AB057099.1), Kazak3 (AB057098.1), USA35 (AB057095.1), Italy329 (AB057094.1), Arizona2 (AB057075.1), Arizona1 (AB057074.1).

Interestingly, the sequences of the EPIYA-D segments in Bhutanese strains showed large variation (Fig. 1) which differed from strains deposited in GenBank where more than 90% of EPIYA-D segments had the same amino acid sequences^26^. Reflecting this variation, pair-wise genetic distances (Kimura-2-parameter model) among Bhutanese East Asian-type cagA were large (median 0.078, mean 0.074, and variance 0.025) with average sequence identity 91.6%. In contrast, the sequences of the EPIYA-C segments in Bhutanese strains were very similar (Fig. 1) and were identical to typical sequences of EPIYA-C segments deposited in GenBank (median, mean, variance of pair-wise genetic distances and average sequence identity among Bhutanese Western-type cagA were 0.01, 0.015, 0.0002, and 98.4%, respectively).

The EPIYA motif in these strains is shown in Table 3. We obtained 11 types of EPIYA or EPIYA-like sequences. In total, 710 EPIYA motifs were obtained from 206 CagA sequences. On average, each CagA sequence contained approximately 3 EPIYA motifs. The 3 most frequent EPIYA motifs were EPIYA (552/710 = 77.8%), EPIYT (14.8%), and ESIYT (6.2%). This result differs from our previous study examining 560 CagA loci deposited in GenBank (92.3% had EPIYA, 5.1% EPIYT, and 0.4% ESIYT)^26^. The EPIYA-B motif had a high degree of variation in the five amino acids (e.g., EPIYA, EPIYT and ESIYT) (Table 3). Among 190 EPIYA-B motifs in East Asian-type CagA, EPIYT was more frequent than EPIYA (53.7% vs. 20.0%). However, there was no association between EPIYA-like sequence type and disease presentation.

### *cagA* genotypes and histological findings

The degree of inflammation (monocyte infiltration), neutrophil activity, atrophy, and intestinal metaplasia were classified into four grades according to the updated Sydney system (0 to 3)^27^. Chronic inflammation and neutrophil activity are characteristic of *H. pylori* infection. Antral predominant gastritis is associated with DU whereas pangastritis with atrophy and intestinal metaplasia is predominant in determining gastric cancer risk^27^.

Individuals with East Asian-type CagA, either single-repeat (ABD) or multiple-repeat (AB’BD and ABBBD) had a slightly higher atrophy score in the antrum than individuals with Western-type CagA (mean score [median score]; 1.45 [1], and 1.43 [1] vs. 1.07 [1]; both P = 0.03), although the statistical differences were disappeared after using multivariate analysis adjusting for age and sex (odds ratios [OR] = 3.30, 95% confidence interval [CI] = 0.61 to 26.39 and OR = 3.79, 95% CI = 0.71 to 28.34). Individuals with the multiple-repeat East Asian-type CagA tended to have higher scores for intestinal metaplasia in the antrum than those with single-repeat East Asian-type CagA (mean [median]; 0.22 [0] vs. 0.11 [0], P = 0.10). Subjects infected with *H. pylori* with the EPIYT motif tended to have higher activity score in the antrum than those infected with EPIYA motif strains (mean [median]; 1.58 [2] vs. 1.37 [1], P = 0.06).

### CagA immunohistochemistry

We performed immunohistochemistry for 205 *H. pylori* culture-positive cases to detect immunoreactivity with CagA and anti-East Asian-type CagA-specific antibody (α-EAS Ab) (Table 4). α-EAS Ab is immunoreactive with only the East Asian-type CagA^28^ and proved useful for typing CagA immunohistochemically in Japan^29^, Vietnam and Thailand^30^. One biopsy specimen for an *H. pylori* culture-positive case (infected with the East Asian-type CagA strain) was unavailable and was excluded from the analyses. Three samples infected with *cagA*-undetermined strains showed negative immunoreactivity with both anti-CagA and α-EAS Abs. The remaining 205 samples showed positive immunoreactivity with the anti-CagA Ab. As expected, all 15 samples infected with Western-type cagA strains showed negative immunoreactivity with α-EAS Ab. Surprisingly, only 68 out of 188 samples (36.2%) infected with East Asian-type CagA strains tested positive for α-EAS Ab. The target α-EAS sequences in EPIYA segment D (Fig. 1) was designed based on the sequences of Japanese strains^28^. Only 6 Bhutanese strains had identical sequences for the α-EAS Ab designed amino acid sequence and showed positive immunoreactivity. When one or two amino acid sequences were different from the designed amino acid sequences (70 cases), positive α-EAS Ab results were detected in 72.9% of cases (51/70). When more than three amino acid sequences were different from the designed amino acid sequence (112 cases), positive α-EAS Ab results decreased to 11.6% of cases (13/112). Using PCR-based sequencing as the gold standard, the sensitivity, specificity, negative predictive value, and positive predictive value of the α-EAS Ab were 36.2%, 94.1%, 11.8%, and 98.6%, respectively in Bhutan. Overall accuracy rate was only 41%. Therefore, the α-EAS Ab was not useful to distinguish East Asian-type CagA strains from other CagA strains in the Bhutanese population. There were no definite relationship between the rate of α-EAS Ab positivity and clinical presentation (42.6% [69/162] for gastritis, 42.9% [9/21] for GU, 47.4% [9/19] for DU and 100% [1/1] for gastric cancer).

### *vacA* genotypes

**Figure 2:**
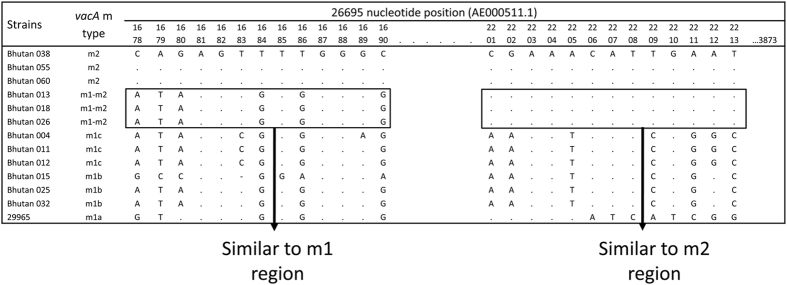
The *vacA* m1-m2 chimeric genotypes. Approximately 85% of the gene sequences for m1-m2 chimeric genotypes were similar to *vacA* m1, and the remaining nucleotides were similar to *vacA* m2.

All 209 strains had the *vacA* s1 genotype. PCR results showed that 80 strains (38.3%) possessed the *vacA* m1 genotype and 42 strains (20.1%) possessed the m2 genotype. The genotype of the remaining 87 strains could not be distinguished based on the length of the PCR product. Therefore, we examined the *vacA* genotypes by DNA sequencing. A total of 205 strains were successfully sequenced. The m1 type can be further subdivided into m1a, m1b, and m1c^6^. Among 80 Bhutanese m1 strains, 46 were classified as m1b, 34 as m1c, and none as m1a. Forty-two strains were classified as m2. Interestingly, 83 strains could not be typed based on PCR and were located on a branch between m1 and m2. These strains contained special sequences with m1-m2 chimeric patterns (Fig. 2). Around 85% of the gene by length was consistent with the *vacA* m1 sequence, and the remaining portion was similar to *vacA* m2. The predominant East Asian-type cagA had the *vacA* s1 m1-m2 chimeric genotype (80/209, 38.3%) or s1m1 (69/209, 33.0%). Only one Western-type *cagA* strain had the s1 m1-m2 chimeric genotype. There was no relationship between *vacA* and *cagA* types in Bhutan (P = 0.65).

### *vacA* genotypes and histological findings

**Figure 3:**
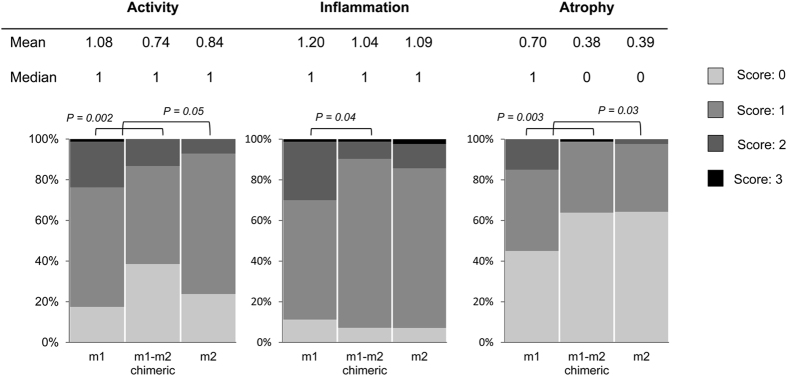
The *vacA* m region genotypes and histological findings in the corpus. Subjects infected with the *vacA* m1 genotype showed higher mucosal atrophy score than those with m2 genotype. Subjects infected with *vacA* m1 genotype also had higher histological severity scores than those with m1-m2 chimeric genotypes.

Histological findings of *vacA* m genotypes showed that subjects infected with m1 strains had a higher score of corporal mucosal atrophy than those with m2 genotypes (mean score [median score]; 0.70 [1] vs. 0.39 [0], P = 0.03) (Fig. 3). Subjects infected with *vacA* m1 genotypes also had a higher scores of corporal activity, inflammation, and atrophy than those with m1-m2 chimeric genotypes (mean [median]; 1.08 [1] vs. 0.74 [1], 1.20 [1] vs. 1.04 [1], and 0.70 [1] vs. 0.38 [0], P = 0.002, P = 0.04, and P = 0.003, respectively) (Fig. 3). Subjects infected with *vacA* m1 also had a significantly higher risk of activity and atrophy in the corpus after adjusting for age and sex (OR = 4.14, 95% CI = 1.87 to 9.17 and OR = 2.74, 95% CI = 1.39 to 5.42, respectively) than those with the m1-m2 chimeric type. However, there were no significant differences in histological scores between *vacA* m1-m2 chimeric type and m2.

### Population structure and phylogenetic position

**Figure 4:**
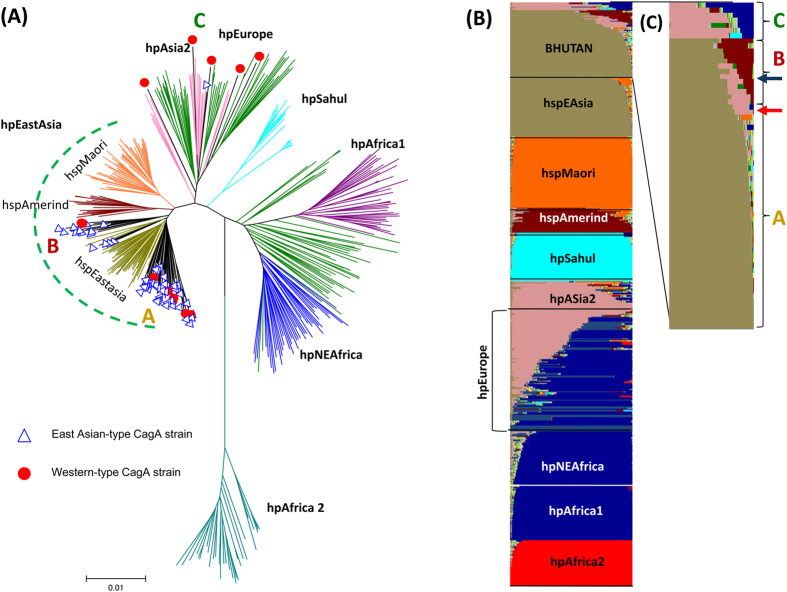
MLST phylogeny and population structure of Bhutanese strains. The population types of 13 Western-type CagA and 50 East Asian-type CagA strains were analyzed by MLST. The strains belonged to three groups: the main branch included hspEAsia strains (group **A**) and was between hspEAsia and hspAmerind (group **B**) and hpEurope/hpAsia2 strains (group **C**) (Fig. 4A). An MLST analysis revealed that most Western-type CagA strains belonged to sub-branch group A or C (red dots). In contrast, only one East Asian-type CagA strain belonged to group C (blue triangle). Figure 4B,C shows the results of a STRUCTURE analysis assuming K = 15, which had the highest posterior probability of the five runs. Each vertical line of the bar chart represents a single strain, and the line colors indicate populations to which the strain may belong. The lengths of the colors in a line are proportional to the probabilities that the strain belongs to the particular population. When the bars were magnified and aligned from top to botom in descending order with respect to the dark yellow color, only two strains were not concordant between the phylogeny and population structure analyses (blue arrow [group A in the phylogeny] in opposite with red arrow) (Fig. 4C).

Concordant with the evidence that the Bhutanese strains contained virulent *H. pylori* genotypes (East Asian-type CagA and *vacA* s1), population analyses showed that the main branch belonged to the hspEAsia strains (blue triangle) (Fig. 4A: group A). Interestingly some East Asian-type CagA strains were located between the hspEAsia and hspAmerind (group B). The AB’BD and ABBD-type CagA was evenly distributed among groups A and B (Table 5).

To confirm these results, we investigated the population structure of the Bhutanese strains using the highest posterior probability of the five runs (K = 15) by STRUCTURE. Consistent with MLST phylogeny, the Bhutanese strains showed the most color commonality with hspEAsia strains (dark yellow) and some of which shared components with hspAmerind (dark red color) which might represent an intermediate evolutionary stage between *H. pylori* from European and East Asian countries. The hspAmerind subgroup of hpEastAsia was isolated from Inuits and Amerinds in North and South America^21^. Only the results for two strains were not concordant between phylogenetic and population structure analyses; however both shared an hspEAsia component (blue arrow [group A in the phylogeny] in opposite with red arrow). Group C had a high proportion of m2 and no m1-m2 chimeric types, which suggests a little recombination in the hpEurope Bhutanese strains (Table 5). However, all m1-m2 chimeric type strains were categorized as group A (80%, 12/15) or B (20%, 3/15).

### Phylogenetic origin and histological findings

Subjects infected with hspEAsia strains (group A) had a significantly higher inflammation score in the antrum than those with hpEurope strains (group C) (mean score [median score]; 1.91 [2] vs. 1.33 [1], P = 0.03). Subjects infected with hspEAsia had a significantly higher risk of inflammation in the antrum after adjusting for age and sex (OR = 6.10, 95% CI = 1.00 to 38.13) than those with the hpEurope strains. Moreover, those infected by group A strains tended to have more inflammation than those infected by group B strains (mean [median]; 1.91 [2] vs. 1.55 [2], P = 0.08).

### Nucleotide and amino acid sequences

All nucleotide sequence data are available under the DDBJ accession numbers LC067575–LC068428.

## Discussion

We confirmed that almost all Bhutanese *H. pylori* strains possessed *cagA* of the East Asian-type CagA type, which is associated with a more robust mucosal inflammatory reaction than Western-type CagA^14^,^15^,^16^. Histological analsysis of gastric mucosa from Bhutanese showed that infection with East Asian-type CagA (AB’BD and AB’BBD) tended to increase the risk of atrophy compared to those infected with Western-type CagA strains. Therefore, in addition to host and environmental factors, the presence of a CagA type associated with an increased inflammatory response might in part be responsible for the higher risk of gastric cancer in Bhutan than in India.

Intriguingly, the very rare AB’BD CagA type was dominant among East Asian-type CagA (51.3%) in Bhutan. There are data that the biological activity of CagA is related in part to the number of CagA phosphorylation sites. *In vitro* studies have suggested that phosphorylation of EPIYA-C is necessary, but not sufficient, to induce the epithelial cell elongation *in vitro*, a morphology was originally referred to as the “hummingbird phenotype” which reflects changes in host cell signaling pathways^31^. It contributes to the epithelial proliferation and pro-inflammatory processes as well as the disruption of cell-to-cell junctions, or loss of cell polarity, all of which are seen in gastric cancer^32^. A previous study^31^ showed that EPIYA-B is also important because at least two phosphorylated EPIYAs are necessary for the elongation phenotype. It was found that East Asian *H. pylori* expressing CagA EPIYA-A/EPIYA-D or EPIYA-B/EPIYA-D, but not EPIYA-A/EPIYA-B, induced moderate epithelial cell elongation^31^. In the case of Western-type CagA, the incidence of gastric cancer was higher in patients infected with strains carrying multiple EPIYA-C repeats than in those infected with strains with a single repeat^9^,^10^,^26^,^33^,^34^. However, there are considerable data to suggest that multiple EPIYA-C repeats are a response to atrophic gastritis with hypochlorhydria rather than its cause^10^. Because gastric cancer is associated with atrophic gastritis and hypochlorhydria, it is not surprising that even in Japanese individuals infected with East Asian-type CagA strains, most strains with multiple EPIYA-B repeats are isolated from patients with gastric cancer^9^. Therefore, the high prevalence of Bhutanese strains with CagA containing multiple EPIYA-B segments is consistent with a high prevalence of atrophic gastritis in Bhutan. Based on pepsinogen levels, the Bhutanese population has a higher incidence of advanced mucosal atrophy, even in the younger population, than other populations (e.g., Japan, Singapore, and the USA)^35^.

Interestingly, the structure of East Asian-type CagA in Bhutan differed from the typical East Asian-type CagA. In particular, the first 18 amino acids of EPIYA-D had what appears to be a population-specific variation in Bhutan which was responsible for the low accuracy of α-EAS Ab. Furthermore, the variance in EPIYA motifs also differed. In Bhutan, EPIYA (77.8%) was the predominant type, followed by EPIYT (14.8%) and ESIYT (6.2%). Previous studies reported that EPIYT was the second most common EPIYA-B sequence of Western-type *cagA*, but was very rare in East Asian-type *cagA*^14^,^26^,^36^. In our previous study of 1,796 EPIYA motifs, including 274 Western and 286 East Asian strains, found 92.2% were EPIYA. EPIYT and ESIYT were only found in 5.1% and 0.3% of strains, respectively^26^. Zhang *et al.* analyzed 364 Western-type *cagA* strains and reported that gastric cancer was significantly associated with EPIYA sequences compared with gastritis alone, whereas EPIYT sequences was significantly associated with DU^36^. The role of the EPIYT-B motif in East Asian-type CagA remains unclear and future studies are necessary to determine whether specific CagA sequences are involved in the pathogenesis of Hp-associated disease.

In Bhutan, all strains had the *vacA* s1 genotype, but the *vacA* m region could not be distinguished for 83 samples using PCR because they contained m1-m2 chimeric sequences. Although the prevalence of the *vacA* m1-m2 chimeric genotype tended to be higher in strains obtained from GU patients than that in those obtained from DU patients, the sample sizes of those with ulcers were small and further studies are needed to evaluate the significance of this observation. m1-m2 chimeric genotype have been rarely found^37^,^38^,^39^. Bhutanese strains possessed specific genotypes for both *cagA* and *vacA* that are very rare in other countries. *In vitro* level of vacuolating activity in strains with the m1-m2 chimeric genotype have been reported to be comparable to those of m1 strains, and were higher than those of m2 strains^39^. In contrast, histological findings showed that *vacA* m1 genotypes was associated with higher activity, inflammation, and atrophy than m1-m2 chimeric genotypes in Bhutan. Further studies are necessary to clarify the whether there is biological importance of the m1-m2 chimeric type.

The Bhutanese strains with East Asian-type CagA were primarily categorized as the hpEAsia population type which had severer histological scores than other populations in this study. This association is consistent with the ASR of gastric cancer in the East Asia region (24.2/100.000) being greater than in the European continent and the South-Central Asia region (9.4 and 6.7/100,000, respectively). Interestingly some also shared an hspAmerind component based on population genetic analyses suggesting that Bhutanese share part of lineage of hspAmerind ancestry. A previous human DNA study concluded that the Africans being the first group of people from which the rest of the human populations split are most divergent from other human populations^40^,^41^. The second major split separated the North Eurasian supercluster (Caucasians, Northeast Asians, and Amerindians) from the Southeast Asian supercluster (Southeast Asians, Australians, Papua New Guineans, and Pacific Islanders)^42^. Amerindians and the current Northeast Asians (Tibetans, Koreans, Japanese, and Mongolians) separated before Amerindians migrated to North America^43^. Although far less likely, an alternative hypothesis is that hspAmerind arose after crossing the Bering Strait, and shared ancestry with the Bhutan population. Further analyses are necessary to confirm the origin of the Bhutan strains. Interestingly, Furuta *et al.* have suggested that Amerind CagA could be intermediate between Western- and East Asian-type CagA; segment C of Amerind CagA contained part of segment D^44^. This remains highly speculative.

The data suggest that Bhutanese strains were influenced by contact with or gene flow from India where some strains are hpEurope^45^,^46^. There are many ethnic groups in Bhutan; however most people included in this study are major ethnic groups “Ngalops” and “Sharchops”; the “Ngalops” are people of Tibetan origin, and the “Sharchops” are the population of mixed Tibetan and Southeast Asian descent. It remains possible that some Bhutanese and Indian strains share a common ancestor.

In conclusion, the higher prevalence of *H. pylori*-associated atrophic gastritis in Bhutanese is consistent with a higher risk of gastric cancer and the high prevalence of the more virulent East Asian-type CagA. The presence of a very rare and population-specific sequence in Bhutanese strains might represent a turning point in the evolution of *H. pylori* and help explain differences between strains from European countries and those from East Asian countries.

## Materials and Methods

### Patients and *H. pylori*

*H. pylori* strains were obtained from the gastric mucosa of Bhutanese volunteers who underwent endoscopy in December 2010 in three cities in Bhutan (Thimphu, Punakha, and Wangdue), as described in our previous study^47^. Presentations for *H. pylori*-infected subjects included gastritis, DU, GU, and gastric cancer. DU, GU, and gastric cancer were identified by endoscopy, and gastric cancer was further confirmed by histopathology. Experienced endoscopists (RV, TR, LT, VM and YY) performed all endoscopy procedures and determined the clinical data. Written informed consent was obtained from all participants, and the protocol was approved by the Ethics Committees of Jigme Dorji Wangchuck National Referral Hospital, Bhutan, Chulalongkorn University, Thailand, and Oita University Faculty of Medicine, Japan. We declare that all procedures contributing to this work comply with the ethical standards of the relevant national and institutional committees on human experimentation and with the Helsinki Declaration of 1975, as revised in 2008.

### Isolation and genotyping of *H. pylori*

*H. pylori* colonies were cultured from antral biopsy specimens using standard methods^37^. *H. pylori* DNA was extracted from these colonies for genotyping using the QIAamp DNA Mini Kit (QIAGEN, Valencia, CA, USA) according to the manufacturer’s instructions. The *cagA* status was determined by PCR amplification and direct sequencing of the 3′ repeat region of *cagA*, as described previously^11^. The absence of *cagA* was confirmed by the presence of a *cag* PAI empty site, as described previously^48^. The EPIYA segment types of CagA were compared with previous data obtained from GenBank using the program WebLogo (version 3) (http://weblogo.threeplusone.com/).

The *vacA* genotyping (s1a, s1b, s1c, or s2; and m1a, m1b, m1c, or m2) was performed following previously described methods^6^,^10^,^49^. Genetic distances for the *vacA* m region were estimated by the six-parameter method, and phylogenetic trees were constructed using the neighbor-joining method including 112 previously published *vacA* m region sequences as a reference^50^. DNA sequencing was performed using the Big Dye Terminator v3.1 Cycle Sequencing Kit on an AB 3130 Genetic Analyzer (Applied Biosystems, Foster City, CA) according to the manufacturer’s instructions. Multiple sequence alignments of the vacA sequences were generated using MAFFT version 7 (available in http://mafft.cbrc.jp/alignment/server/) and confirmed by visual inspection.

### Population and phylogenetic analysis based on MLST data

Thirteen Bhutan strains possessing the Western-type CagA and 50 Bhutan strains possessing the East Asian-type CagA were randomly selected and seven MLST genes were sequenced in each of these strains by PCR-based sequencing as previously described^51^. Additionally, MLST sequence data were downloaded from the PubMLST database (http://pubmlst.org/) and 430 strains that are representative of each *H. pylori* population were chosen. Then, the Bhutan and selected PubMLST data were combined and the population type of the Bhutanese strains was analyzed using the population analysis software STRUCTURE (v.2.3.2)^52^. Markov chain Monte Carlo (MCMC) simulations were run in STRUCTURE using the admixture model with a burn-in of 20,000, followed by 30,000 iterations for each run. The analysis was repeated for a population number (K) ranging from 7 to 15, and 5 runs were performed for each K. For a given K, STRUCTURE determines K population components, which are represented using K colors. A neighbor-joining tree (Kimura’s two-parameter model) was constructed using the same dataset.

### Histology and immunohistochemistry

Biopsy specimens were collected from the antrum and corpus and fixed in 10% buffered formalin for 24 h and then embedded in paraffin. Immunohistochemistry to determine CagA status and the status of East Asian-type CagA was performed as described previously^28^. Briefly, after antigen retrieval and inactivation of endogenous peroxidase activity, tissue sections were incubated with α-*H. pylori* antibody (DAKO, Glostrup, Denmark), anti-CagA antibody (b-300; Santa Cruz Biotechnology, Santa Cruz, CA, USA) or α-EAS Ab diluted 1:2,000 with diluting solution (DAKO) overnight at 4 °C. After washing, the sections were incubated with biotinylated goat anti-rabbit or anti-rat IgG (Nichirei Co., Tokyo, Japan), followed by incubation with a solution of avidin-conjugated horseradish peroxidase (Vectastain Elite ABC kit; Vector Laboratories Inc., Burlingame, CA, USA). Peroxidase activity was detected using H_2_O_2_/diaminobenzidine substrate solution. *H. pylori* were identified by Giemsa staining and positively immunostained with anti-*H. pylori* antibody. The degree of inflammation, neutrophil activity, atrophy, intestinal metaplasia, and bacterial density were classified into four grades according to the updated Sydney system: 0, ‘normal’; 1, ‘mild’; 2, ‘moderate’; and 3, ‘marked’^27^. Bacterial loads greater than or equal to grade 1 were considered positive for *H. pylori*. Samples classified as grade 1 or higher were considered atrophy-positive^53^. The bacterial load was classified into four grades: 0, ‘none’; 1, ‘mild’; 2, ‘moderate’; and 3, ‘marked’ based on specimens stained with May-Giemsa^27^.

### Statistical analysis

Discrete variables were tested using the chi-square test and Fisher’s exact probability test (average age and sex ratio vs. diagnosis, relationship between genotypes); The difference of histologic score between genotypes were tested using the Mann-Whitney *U* test. A multivariate logistic regression model was used to calculate the OR and 95% CI of the clinical presentations including age, sex, and *H. pylori* genotype. All determinants with P-values of less than 0.10 were combined in the full model for the logistic regression, and the model was reduced by excluding variables with P-values of greater than 0.10. A P-value of < 0.05 was accepted as statistically significant. SPSS statistical software package version 19.0 (SPSS, Inc., Chicago, IL, USA) was used for all statistical analyses.

## Additional Information

**How to cite this article**: Matsunari, O. *et al.* Rare *Helicobacter pylori* Virulence Genotypes in Bhutan. *Sci. Rep.* **6**, 22584; doi: 10.1038/srep22584 (2016).

## Figures and Tables

**Table 1:** Association between *Helicobacter pylori* virulence factors and clinical presentations.

	Total		Gastritis		Gastric ulcer		Duodenal ulcer		Gastric cancer		Unclear diagnosis	
	n = 209	%	n = 165	%	n = 21	%	n = 19	%	n = 1	%	n = 3	%
*cagA* positive	206	(98.6)	162	(98.2)	21	(100)	19	(100)	1	(100)	3	(100)
*cagA* undetermined	3	(1.4)	3	(1.8)	0	(0.0)	0	(0.0)	0	(0.0)	0	(0.0)
*cagA* genotype undetermined	2	(1.0)	2	(1.2)	0	(0.0)	0	(0.0)	0	(0.0)	0	(0.0)
East Asian-type cagA	189	(91.7)	149	(90.1)	18	(85.7)	18	(94.7)	1	(100)	3	(100)
Western-type cagA	15	(7.3)	11	(6.8)	3	(14.3)	1	(5.3)	0	(0.0)	0	(0.0)
*vacA* s1	209	(100)	165	(100)	21	(100)	19	(100)	1	(100)	3	(100)
s2	0	(0.0)	0	(0.0)	0	(0.0)	0	(0.0)	0	(0.0)	0	(0.0)
*vacA* m1	80	(38.3)	63	(38.2)	5	(23.8)	9	(47.4)	1	(100)	2	(66.7)
m2	42	(20.1)	32	(19.4)	4	(19.0)	5	(26.3)	0	(0.0)	1	(33.3)
m1m2 chimera (m12)	83	(39.7)	66	(40.0)	12	(57.1)	5	(26.3)	0	(100)	0	(14.3)
m genotype undetermined	4	(1.9)	4	(2.4)	0	(0.0)	0	(0.0)	0	(0.0)	0	(0.0)
East Asian cagA/*vacA* s1m1	69	(33.0)	54	(32.7)	3	(14.3)	9	(47.4)	1	(100)	2	(66.7)
East Asian cagA/*vacA* s1m2	37	(17.7)	28	(17.0)	4	(19.0)	4	(21.1)	0	(0.0)	1	(33.3)
East Asian cagA/*vacA* s1m12	80	(38.3)	64	(38.8)	11	(52.4)	5	(26.3)	0	(0.0)	0	(0.0)
Western cagA/*vacA* s1m1	8	(3.3)	6	(3.7)	2	(9.5)	0	(0.0)	0	(0.0)	0	(0.0)
Western cagA/*vacA* s1m2	5	(2.4)	4	(2.5)	0	(0.0)	1	(5.3)	0	(0.0)	0	(0.0)
Western cagA/*vacA* s1m12	1	(0.5)	0	(0.0)	1	(0.48)	0	(0.0)	0	(0.0)	0	(0.0)
Others	9	(4.3)	9	(4.3)	0	(0.0)	0	(0.0)	0	(0.0)	0	(0.0)

Others: *cagA* genotype undetermined and/or *vacA* m genotype undetermined.

**Table 2:** Association between EPIYA segment type of CagA and clinical presentations.

	Total	Gastritis	Gastric ulcer	Duodenal ulcer	Gastric cancer	others
Western-type CagA						
AB*	1	1	0	0	0	0
AC	1	1	0	0	0	0
ABC	13	9	3	1	0	0
Total	15	11	3	1	0	0
East Asian-type CagA						
ABD	82	68	5	7	1	1
AB’D	1	1	0	0	0	0
ABBD	5	5	0	0	0	0
AB’BD	97	72	13	10	0	2
AB’B’BD	2	2	0	0	0	0
BD	2	1	0	1	0	0
Total	189	149	18	18	1	3

^*^AB type was defined as Western-type CagA since the sequence of the B segment was mostly identical with the Western-type B segment (TGQVASPEEPIYAQVAKKVKAKIDRLDQIASGLGGVGQAG).

**Table 3:** Frequencies of the 11 EPIYA motif types.

	All motifs		A motif		B’ motif		B motif		C or D motif	
All CagA types	**EPIYA**	**552**	**EPIYA**	**201**	**EPIYA**	**99**	**EPIYA**	**50**	**EPIYA**	**202**
	EPIYT	105			EPIYT	2	EPIYT	103		
	ESIYT	44					ESIYT	44		
	ESIYA	2					ESIYA	2		
	EYIYA	1					EYIYA	1		
	ETIYT	1					ETIYT	1		
	KSIYT	1					KSIYT	1		
	EPIYV	1					EPIYV	1		
	EPIYS	1					EPIYS	1		
	GPIYA	1	GPIYA	1						
	EPLYA	1							EPLYA	1
Total		710		202		101		204		203
Western-type CagA	**EPIYA**	**41**	**EPIYA**	**15**			**EPIYA**	**12**	**EPIYA**	**14**
	EPIYT	1					EPIYT	1		
	EPIYS	1					EPIYS	1		
Total		43		15				14		14
East-Asian-type CagA	**EPIYA**	**511**	**EPIYA**	**186**	**EPIYA**	**99**	**EPIYA**	**38**	**EPIYA**	**188**
	EPIYT	104			EPIYT	2	EPIYT	102		
	ESIYT	44					ESIYT	44		
	ESIYA	2					ESIYA	2		
	EYIYA	1					EYIYA	1		
	ETIYT	1					ETIYT	1		
	KSIYT	1					KSIYT	1		
	EPIYV	1					EPIYV	1		
	GPIYA	1	GPIYA	1						
	EPLYA	1							EPLYA	1
Total		667		187		101		190		189

**Table 4:** *cagA* genotypes and CagA immunoreactivity.

	anti-CagA Ab-positive	α-EAS Ab-positive
*cagA* positive (n = 205)	205 (100%)	
*cagA* undetermined (n = 3)	0 (0%)	0 (0%)
*cagA* genotype undetermined (n = 2)	2 (100%)	1 (50%)
East Asian-type *cagA* (n = 188)	188 (100%)	68 (36.2%)
Western-type cagA (n = 15)	15 (100%)	0 (0%)

**Table 5:** Association between MLST phylogeny type and *cagA* and *vacA* type.

Group	n	AB’BD	ABD	Western-type CagA	others	m1	m2	hybrid
A	46	17 (73.9%)	18 (85.7%)	6 (46.2%)	5 (83.3%)	20 (74.1%)	13 (61.9%)	13 (86.7%)
B	11	5 (21.7%)	3 (14.3%)	2 (15.4%)	1 (16.7%)	5 (18.5%)	4 (19.0%)	2 (13.3%)
C	6	1 (4.3%)	0 (0.0%)	5 (38.5%)	0 (0.0%)	2 (7.4%)	4 (19.0%)	0 (0.0%)
Total	63	23 (100.0%)	21 (100.0%)	13 (100.0%)	6 (100.0%)	27 (100%)	21 (100.0%)	15 (100.0%)
